# COVID-19 vaccine short-term adverse events in the real-life family practice in Krakow, Poland

**DOI:** 10.1080/13814788.2022.2147500

**Published:** 2022-12-05

**Authors:** Marek Oleszczyk, Zuzanna Marciniak, Katarzyna Nessler, Ewa Wójtowicz, Nataliya Szozda, Elżbieta Kryj-Radziszewska, Maria Boroń, Klaudia Gajos, Mateusz P. Paziewski, Paweł Sajdak, Adam Windak

**Affiliations:** a Department of Family Medicine, Jagiellonian University Medical College, Krakow, Poland; b Family Medicine Student Interest Group, Faculty of Medicine, Jagiellonian University Medical College, Krakow, Poland; c The Ludwik Rydygier Viovodship Hospital, Krakow, Poland; d Department of Family and Community Medicine, University of Toronto/Toronto Western Hospital PGY1, Toronto, Canada; e Independent Public Health Care Center, Myślenice, Poland; f Department of Anatomy, Jagiellonian University Medical College, Krakow, Poland

**Keywords:** COVID-19 vaccine, vaccine adverse event, primary care, family medicine, pragmatic study

## Abstract

**Background:**

In manufacturers’ trials, vaccination against COVID-19 proved to be safe and effective. The officially reported frequency of vaccine adverse events (VAEs) in Poland is lower than that declared by the manufacturers. The anti-vaccination activists questioned the trustworthiness of official data.

**Objectives:**

The aim was to explore the real-life prevalence of VAEs in general practice settings and the factors that may influence it.

**Methods:**

In this pragmatic, mixed prospective and retrospective study, patients vaccinated against COVID-19 between May and October 2021 in three GP practices in Krakow, Poland, were enrolled. Their demographic (age, sex, level of education) and clinical data (weight and height, smoking status, history of allergies, COVID-19 and chronic diseases) were collected. Then, they were interviewed about VAEs they experienced.

**Results:**

Out of 1530 patients invited to participate, 1051 (69%) agreed and were eligible for analyses. Only 8.8% did not report any VAE. Pain at the injection site was the most frequently reported reaction (800, 76.2%). The most prevalent systemic ones were excessive fatigue/lethargy (527, 50.6%), sleep/circadian rhythm disturbances (433, 41.6%) and headache (399, 38.3%). Fifty required medical assistance − 39 experienced presyncope (3.7%) and 11 loss of consciousness (1.1%). Only two others were hospitalised. Females, younger adults, those with higher education and with a history of COVID-19 reported systemic VAEs more frequently, while those who were older and obese were less likely to report local reactions.

**Conclusion:**

Although more than 90% of patients vaccinated against COVID-19 in general practice settings may experience VAEs, in short-term observation, the vast majority are localised and mild.

KEY MESSAGES

- Over 90% of patients vaccinated in real-life family practice experience COVID-19 VAEs but the majority are mild in short-term observations.
- Female sex, younger age, higher level of education and history of COVID-19 correlate with the higher occurrence of systemic VAEs.
- Older and obese patients are more likely to experience local VAEs.

## Introduction

The global spread of SARS-CoV-2 and the pandemic of COVID-19 forced the governments and healthcare systems, including primary care, to adapt quickly to this unprecedented situation [1–3]. The scientific community and pharmaceutical companies made efforts to develop vaccines. In December 2020, supported by the evidence on the first mRNA vaccine (mRNAV) [4], the US Food and Drug Administration gave emergency authorisation to use this vaccine in the general population [5]. Soon, European Medicines Agency took a similar decision [6].

In Poland, four COVID-19 vaccines were available, offered within the national vaccination programme framework. They were mRNAV (Comirnaty^®^ by Pfizer/BioNTech, Spikevax^®^ by Moderna) and adenovirus vector vaccines (VVV) (Vaxzevria^®^ by AstraZeneca and Jcovden^®^ by Janssen/Johnson & Johnson).

The manufacturer of Comirnaty said the most frequent adverse reactions in their clinical studies were: injection site pain (>80%) and swelling (10%), fatigue (>60%), headache (>50%), myalgia (>40%), chills (>30%), arthralgia (>20%) and fever (>10%) [7].

The Jcovden manufacturer stated, based on their studies, that the most common local reaction was injection site pain (48.6%), and systemic reactions were headache (38.9%), fatigue (38.2%), myalgia (33.2%) and nausea (14.2%) [8]. Similar safety information was provided by the manufacturers of Spikevax and Vaxzevria [9,10].

A nationwide COVID-19 vaccination programme was started in Poland on 28 December 2020 and has challenged the efficiency of the vaccine adverse events (VAEs) reporting system [11]. In Poland, there is a compulsory VAEs reporting system. Healthcare professionals are obliged to report VAEs to the State Sanitary Inspection. Then, the National Institute of Public Health (NIPH) publishes reports on COVID-19 VAEs. NIPH, in their first report based on epidemiological surveillance (27 December 2020–26 April 2021), reported <7000 VAEs, of which >85% were mild and only 2% severe. With 10.5 million vaccinations, only <0.1% were reported as associated with any VAE [12].

At the time, anti-vaccination movements intensified their activity, accusing the government and pharmaceutical companies of misinforming and disrupting data on the true prevalence of VAEs. This situation was similar to what was observed globally and could concern primary care physicians, too [13–15]. As a result, <60% of the Polish population has been vaccinated against COVID-19, which is significantly below the European Union average (75%) [16].

The study aimed to explore the prevalence of VAEs in general practice settings and the factors that may influence it. It was designed to answer the following questions: how frequent and severe are short-term VAEs in the adult population vaccinated against COVID-19 in the primary care setting? Is the real-life frequency of VAEs different from that reported by vaccine manufacturers or governmental institutions? What are the patients’ characteristics associated with experienced VAEs?

## Methods

### Study design

This is a pragmatic, mixed prospective and retrospective study on patient self-monitored COVID-19 VAEs. The Jagiellonian University Bioethics Committee approved the study on 21 April 2021 (Opinion No. 1072.6120.71.2021)

### Recruitment and data collection

The participants were recruited between 2 May and 31 October 2021 in public COVID-19 vaccination hubs organised in three GP practices in Krakow, Poland. Right before the vaccination, the trained fieldworkers (medical students) invited the patients to participate. All adults ≥18 years old who were qualified for the vaccination, agreed to participate and gave their informed consent were included in the study.

First, the participants answered questions about their characteristics (age, sex, level of education, professional status, smoking status, history of COVID-19, and chronic medical conditions, with a particular interest in allergies and anaphylactic reactions). Their body weight and height were assessed, and their body mass index (BMI) was calculated. When the fieldworkers recruited a patient receiving their second dose in the two-dose vaccination scheme (Comirnaty, Moderna COVID-19 vaccine, or Vaxzevria), they interviewed them about all VAEs they experienced after the first dose (given 4 weeks earlier). Then, 4–6 days after vaccination all participants were contacted by telephone for a follow-up interview. The patients recruited before their first dose in the two-dose vaccination scheme were contacted again 3–5 weeks later (4–6 days after the second dose).

The list of VAEs was established based on the literature review. The multiple-choice questions included local (injection site pain, erythema or swelling) and systemic symptoms (fever, chills, myalgia, arthralgia, axillary tenderness, headache, ocular problems, seizures, allergic reactions, presyncope, transient loss of consciousness, sleep/circadian rhythm disturbances, excessive fatigue/lethargy, feeling of confusion, heart palpitations or other cardiovascular symptoms, limb discolouration, diarrhoea, vomiting and nausea). In both categories, the participants could add any other symptoms they experienced. For this study, we classified the VAEs as ‘severe’ when they required emergency treatment (such as anaphylactic shock), need for hospital admission or when causing death. All others were classified as ‘mild’.

### Data digitalisation and database processing

Questionnaires were digitalised and transferred to a database by a health-data processing company. Then, we verified data transfer quality by comparing 5% of randomly selected questionnaires with corresponding digital records. Only a few minor and negligible errors were found. Finally, before the statistical analysis started, we checked the database for data validity and conducted database cleaning.

### Statistical analysis

To illustrate respondents’ characteristics and frequency of VAEs, we calculated distributions for categorical data and means with standard deviations for quantitative data. Differences between groups were assessed using Chi-square or t-test for dependent groups (respectively for the type of data). Multivariable logistic regression modelling was used to explore the possible influence of independent variables (sex, age, education, BMI, smoking status, chronic medical conditions, history of allergy, anaphylactic shock, COVID-19 infection, type of vaccine) on the occurrence of each VAE which occurred in at least 5% cases. Similarly, multivariable linear regression modelling was used for the total number of local and systemic VAEs. An alpha level of *p* = 0.05 was accepted as the statistical significance test. As we performed many comparisons (including many measures and VAEs), we corrected the level of statistical significance (with the Holm–Bonferroni method). We used Statistica 13 software (Statsoft Inc.).

## Results

### Participants’ characteristics

Overall, the fieldworkers invited 1530 adults who had received their COVID-19 vaccine, and 1071 (70%) agreed, signed informed consent and answered the questions of the first part of the questionnaire. We collected data on 760 patients vaccinated with Jcovden, 293 with Comirnaty, 17 with Vaxzevria and one with Spikevax. Owing to the small number of Vaxzevria and Spikevax participants, we did not include them in the analysis. We discarded two records from the Jcovden group because of substantial questionnaire gaps. Finally, we included data from 1051 (69%) patients: 758 receiving Jcovden and 293 receiving Comirnaty (of whom 231 [78.8%] were recruited when receiving the second dose).

The participants vaccinated with Comirnaty and those with Jcovden differed in their characteristics and comorbidities but not in the history of allergy/anaphylaxis or COVID-19. Table 1 presents the details.

**Table 1.** Participants’ characteristics.

	Total	Pfizer/BioNTech Comirnaty	Janssen/J&J Jcovden	*p*-Value^a^
	*n* = 1051	*n* = 293	*n* = 758	
Age [years] mean (min–max, *SD*)	37.5 (18–85, 14.2)	41.0 (18–83, 16.1)	36.2 (18–85, 13.1)	0.0001
Weight [kg] mean (min–max, *SD*)	75.1 (33–140, 16.4)	75.8 (47–120, 16.2)	74.8 (33–140, 16.5)	0.4423
Height [cm] mean (min–max, *SD*)	172.5 (148–203, 9.7)	170.3 (148–196, 9.2)	173.4 (150–203, 9.8)	0.0000
BMI [kg/m^2^] (min–max, *SD*)	25.1 (13.6–45.2, 4.7)	26.1 (16.9–43.0, 5.1)	24.7 (13.6–45.2, 4.4)	0.0010
	*n* (%)	*n* (%)	*n* (%)	
Sex				
female	493 (47.2%)	176 (60.9%)	317 (41.9%)	0.0000
Level of education				0.0014
elementary	45 (4.3%)	15 (5.1%)	30 (4.0%)	
vocational or middle	477 (45.6%)	104 (35.6%)	373 (49.5%)	
baccalaureate	98 (9.4%)	32 (11.0%)	66 (8.8%)	
higher	426 (40.7%)	141 (48.3%)	285 (37.8%)	
BMI (categories)				0.0000
below normal <18.5	47 (4.5%)	5 (1.7%)	42 (5.6%)	
normal [18.5; 25)	532 (50.9%)	141 (48.6%)	391 (51.7%)	
overweight [25; 30)	307 (29.3%)	78 (26.9%)	229 (30.3%)	
obesity ≥30	160 (15.3%)	66 (22.8%)	94 (12.4%)	
Current smoker	335 (32.0%)	73 (24.9%)	262 (34.7%)	0.0023
Chronic medical conditions	294 (28.0%)	114 (38.9%)	180 (23.7%)	0.0000
AH	118 (11.2%)	49 (16.7%)	69 (9.1%)	0.0005
CVD (other than AH)	33 (3.1%)	15 (5.1%)	18 (2.4%)	0.0221
DM and its complications	41 (3.9%)	25 (8.5%)	16 (2.0%)	0.0000
Thyroid gland abnormalities	69 (6.6%)	36 (12.3%)	33 (4.4%)	0.0000
Asthma/COPD	32 (3.0%)	10 (3.4%)	22 (2.9%)	0.6657
Thrombosis	9 (0.9%)	7 (2.4%)	2 (0.3%)	0.0008
Other chronic conditions^b^	131 (12.5%)	45 (15.4%)	86 (11.3%)	0.0774
No. of chronic conditions				0.0000
0	757 (72.0%)	179 (61.1%)	578 (76.3%)	
1	204 (19.4%)	70 (23.9%)	134 (17.7%)	
2	58 (5.5%)	27 (9.2%)	31 (4.1%)	
3	21 (2.0%)	9 (3.1%)	12 (1.6%)	
≥4	11 (1.0%)	8 (2.7%)	3 (0.4%)	
History of allergy	296 (28.2%)	77 (26.3%)	219 (28.9%)	0.3986
History of anaphylactic shock (before COVID-19 vaccination)	16 (1.5%)	5 (1.7%)	11 (1.5%)	0.7490
History of COVID-19	297 (28.5%)	89 (30.7%)	208 (27.7%)	0.3315

AH: arterial hypertension; BMI: body mass index; COPD: chronic obstructive pulmonary disease; CVD: cardiovascular diseases; DM: diabetes mellitus.

^a^*p*-Value obtained from Chi-square tests and refers to the comparison of both vaccines.

^b^Other chronic conditions included: GI tract (e.g. gastroesophageal reflux disease, chronic gastritis, coeliac disease), musculoskeletal (osteoarthritis, rheumatoid arthritis, gout), ophthalmological (glaucoma, cataract), urological (benign prostate hypertrophy), metabolic and hormonal (e.g. hypercholesterolaemia, hyperprolactinemia) and psychological (e.g. schizophrenia).

### Vaccine adverse events

**Figure 1.**
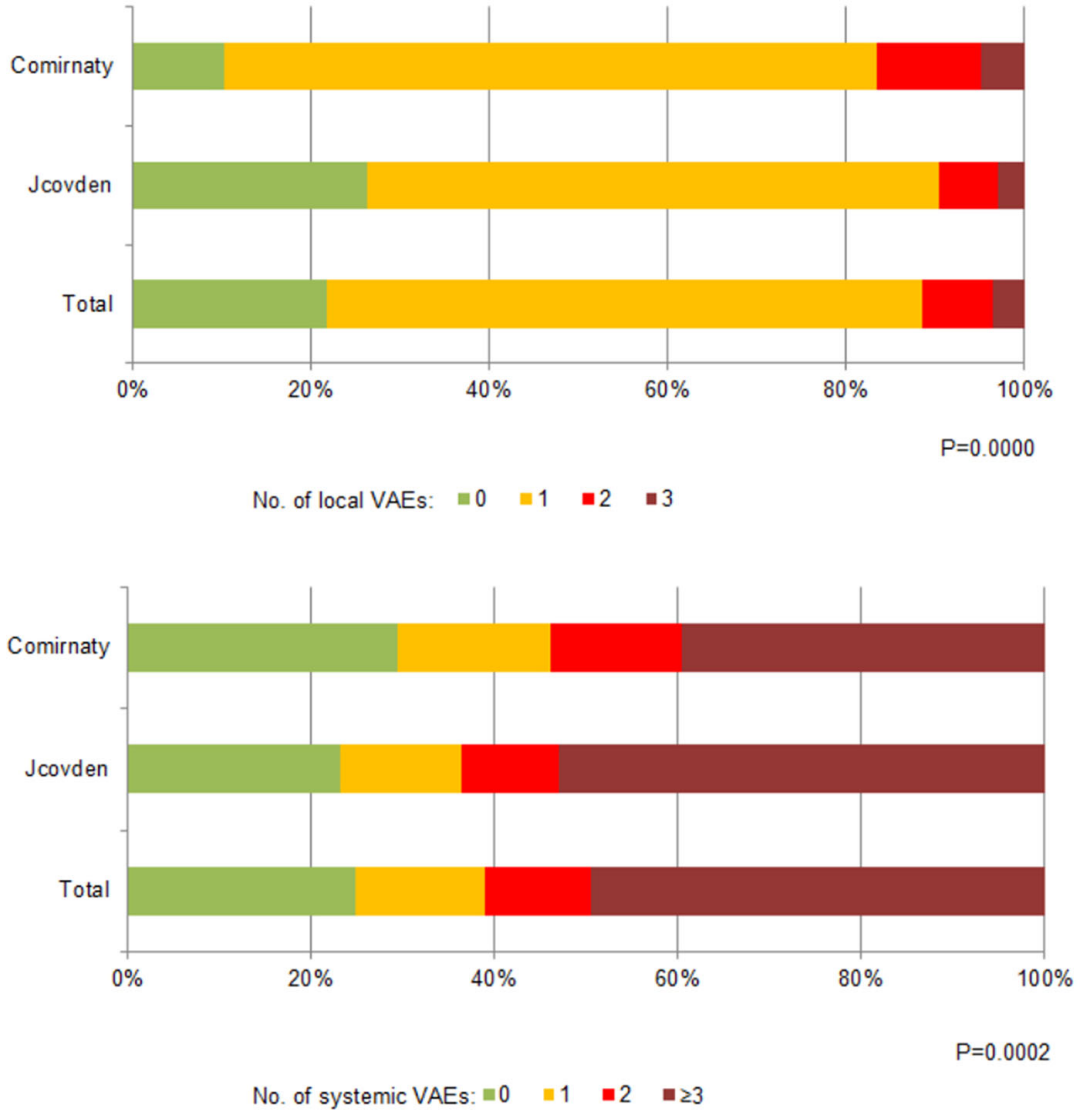
Percentage of participants with local and systemic vaccine adverse events.

Only every eleventh patient (8.8%) had neither local nor systemic reactions (10% in the Jcovden group vs 5.3% in the Comirnaty group), 227 participants reported no localised VAEs (10.2% in the Comirnaty group and 26.3% in the Jcovden group) and 250 participants experienced no systemic reactions (29.4% in the Comirnaty group and 23.2% in the Jcovden group). Table 2 presents the details and Figure 1 presents the percentage of participants experiencing local and generalised VAEs.

**Table 2.** Frequency of local and systemic vaccine adverse events.

		Pfizer/BioNTech Comirnaty				
	Total *n* = 1051	1st dose *n* = 293	2nd dose *n* = 291	Both doses *n* = 293	Janssen/J&J Jcovden *n* = 758	*p*-Value^a^
Local VAE						
Swelling	86 (8.3%)	17 (5.9%)	22 (7.6%)	35 (12.3%)	51 (6.8%)	**0.0037**
Redness	94 (9.1%)	15 (5.2%)	19 (6.6%)	27 (9.5%)	67 (8.9%)	0.7436
Pain	800 (76.2%)	237 (81.2%)	220 (75.6%)	263 (89.8%)	537 (70.9%)	**0.0000**
Systemic VAE						
Fever	240 (22.9%)	5 (1.7%)	17 (5.8%)	19 (6.5%)	221 (29.3%)	**0.0000**
Chills	278 (26.8%)	24 (8.4%)	21 (7.2%)	41 (14.4%)	237 (31.4%)	**0.0000**
Myalgia	363 (34.8%)	40 (13.9%)	58 (19.9%)	83 (29.0%)	280 (36.9%)	**0.0166**
Arthralgia	218 (21.0%)	24 (8.4%)	37 (12.8%)	52 (18.4%)	166 (21.9%)	0.2101
Axillary tenderness	57 (5.5%)	28 (9.9%)	11 (3.8%)	36 (12.8%)	21 (2.8%)	**0.0000**
Headache	399 (38.3%)	56 (19.4%)	56 (19.2%)	88 (30.8%)	311 (41.1%)	**0.0021**
Ocular problems	80 (7.7%)	12 (4.2%)	11 (3.8%)	20 (7.0%)	60 (7.9%)	0.6196
Seizures	27 (2.6%)	3 (1.0%)	1 (0.3%)	4 (1.4%)	23 (3.0%)	0.1437
Allergic reaction	44 (4.2%)	2 (0.7%)	8 (2.8%)	9 (3.2%)	35 (4.6%)	0.2989
Presyncope	39 (3.7%)	2 (0.7%)	4 (1.4%)	5 (1.8%)	34 (4.5%)	0.0380^b^
TLOC	11 (1.1%)	0 (0.0%)	0 (0.0%)	0 (0.0%)	11 (1.5%)	0.0408^b^
Sleep/circadian rhythm disturbances	433 (41.6%)	72 (25.1%)	90 (31.0%)	123 (43.2%)	310 (41.0%)	0.5298
Excessive fatigue/lethargy	527 (50.6%)	88 (30.7%)	92 (31.6%)	133 (46.5%)	394 (52.1%)	0.1058
Feeling of confusion	148 (14.2%)	13 (4.5%)	26 (8.9%)	35 (12.3%)	113 (14.9%)	0.2752
Heart palpitation or other cardiovascular symptoms	40 (3.8%)	6 (2.1%)	5 (1.7%)	12 (4.2%)	28 (3.7%)	0.7044
Diarrhoea	20 (1.9%)	0 (0.0%)	6 (2.1%)	6 (2.1%)	14 (1.9%)	0.7849
Limb(s) discolouration	11 (1.1%)	1 (0.3%)	2 (0.7%)	3 (1.1%)	8 (1.1%)	0.9990
Vomiting	12 (1.2%)	2 (0.7%)	1 (0.3%)	3 (1.1%)	9 (1.2%)	0.8542
Nausea	57 (5.5%)	6 (2.1%)	10 (3.4%)	15 (5.3%)	42 (5.5%)	0.8663
Other systemic reactions^c^	152 (14.5%)	12 (4.1%)	30 (2.9%)	39 (13.4%)	113 (14.9%)	0.5351

VAE: vaccine adverse event; TLOC: transient loss of consciousness.

Statistically significant correlations are set in bold.

^a^*p*-Value obtained from Chi-square tests, refers to the comparisons of both vaccines.

^b^Statistically insignificant after the Holm–Bonferroni correction.

^c^Included episodic, difficult to classify symptoms, e.g. preterm menstrual bleeding, oversensitive skin, generalised or localised (but distant to vaccinated limb, e.g. foot) tingling, better mood, sweats, sensation of cold, anxiety, painful breast, itching of the ears, sore throat, sneezing.

Only 50 participants reported VAEs that required immediate medical assistance: 39 (3.7%) experienced presyncope (all in persons without a history of anaphylactic shock) and only 11 (1.1%) reported transient loss of consciousness after receiving a vaccine (and although there was a statistically significant correlation with a history of anaphylactic shock [*p* = 0.04], this finding cannot be generalised). In two cases we had reliable information about the hospital admission after vaccination (one because of nausea and vomiting, one because of tonsilitis; both in the VVV group).

### Factors associated with VAEs

The logistic regression modelling identified factors correlated with reported VAEs. Female sex and history of COVID-19 were the most frequently positively correlated with systemic VAEs, while older age correlated negatively with most of them. Patients vaccinated with Comirnaty were less likely to have systemic VAEs, with axillary tenderness as the exemption. Tables 3 and 4 present statistically relevant correlations between patients’ characteristics or type of vaccine and VAEs. With the linear regression modelling, we also identified predictors of the number of local (Supplementary Table 1) and systemic (Supplementary Table 2) VAEs. However, those models poorly explain the variability (corrected R2 coefficients are 0.07 and 0.12 for local and systemic VAEs, respectively).

**Table 3.** Variables correlated with local vaccine adverse events in the logistic regression model.

	Adjusted OR (95% CI) of a local reaction	
	VAE	
Participants’ characteristics or type of vaccine	Redness	Pain
Age (ref. the lowest value, yearly)	0.98* **(0.96–0.99)**	0.96*** **(0.95–0.98)**
Obesity (ref. normal BMI)	1.03 (0.51 − 2.04)	0.58* **(0.36–0.91)**
Sex: female (ref. male)	1.75* **(1.08–2.82)**	1.32 (0.94–1.84)
Chronic medical conditions (any) (ref. no chronic conditions)	1.91* **(1.15–3.19)**	1.55* **(1.03–2.32)**
History of COVID-19 (ref. no history COVID-19)	1.28 (0.79–2.07)	1.65** **(1.14–2.39)**
Type of vaccine: Comirnaty (ref. Jcovden)	0.81 (0.47–1.37)	4.43*** **(2.80–7.0)**

Statistically significant correlations are set in bold; *p-value <0.05; **p-value ≤0.01; ***p-value ≤0.001.

BMI: body mass index, CI: confidence interval, OR: odds ratio, VAE: vaccine adverse event.

There was no statically relevant correlation of any variables and swelling. This table includes only the variables with at least one statistically relevant correlation.

**Table 4.** Variables correlated with systemic vaccine adverse events in the logistic regression model.

		Adjusted OR (95% CI) of a systemic reaction										
VAE Participants’ characteristics or type of vaccine		Fever	Chills	Myalgia	Arthralgia	Axillary tenderness	Headache	Ocular problems	Sleep/circadian rhythm disturbances	Excessive fatigue/lethargy	Feeling of confusion	Nausea
Age (ref. the lowest value, yearly)		0.97*** **(0.96–0.99)**	0.96*** **(0.95–0.98)**	0.97*** **(0.96–0.99)**	0.98** **(0.96–0.99)**	0.97* **(0.94–0.99)**	0.97*** **(0.96–0. 98)**	0.99 (0.97–1.01)	0.98*** **(0.97–0.99)**	0.98** **(0.97–0.99)**	0.99 (0.97–1.01)	0.98 (0.96–1.01)
Current smoker (ref. non-smoker)		0.99 (0.7–1.39)	0.82 (0.59–1.14)	0.66** **(0.49–0.9)**	0.75 (0.52–1.07)	1.15 (0.6–2.18)	0.69* **(0.51–0.93)**	0.78 (0.45–1.35)	0.82 (0.61–1.09)	0.75* **(0.57–0.99)**	0.72 (0.47–1.1)	0.71 (0.37–1.36)
Sex: female (ref. male)		1.62** **(1.16–2.27)**	1.71*** **(1.24–2.36)**	1.42* **(1.06–1.9)**	1.42* **(1.01–2.0)**	1.31 (0.7–2.43)	1.56** **(1.17–2.08)**	1.51 (0.91–2.50)	1.34* **(1.01–1.77)**	1.38* **(1.05–1.82)**	1.39 (0.94–2.06)	2* **(1.09–3.69)**
Level of education (ref. Elementary)	Higher	1.06 (0.46–2.43)	1.91 0.79–4.62	2.0 (0.91–4.42)	5.23* **(1.22–22.48)**	2.38 (0.3–18.93)	1.12 (0.56–2.23)	1.08 (0.31–3.77)	1.44 (0.73–2.84)	1.48 (0.77–2.85)	1.72 (0.64–4.61)	0.78 (0.17–3.58)
	Baccalaureate	1.25 (0.5–3.14)	1.36 (0.52–3.6)	1.76 (0.73–4.24)	4.86* **(1.07–22.18)**	4.81 (0.57–40.82)	0.87 (0.4–1.91)	1.41 (0.36–5.49)	0.67 (0.3–1.47)	0.85 (0.40–1.81)	0.79 (0.25–2.5)	1.38 (0.27–6.96)
	Vocational or middle	1.19 (0.52–2.71)	2.18 (0.9–5.24)	1.1 (0.88–4.3)	5.9* **(1.37–25.33)**	3.14 (0.39–25.31)	0.92 (0.47–1.83)	0.91 (0.26–3.19)	1.03 (0.52–2.03)	0.92 (0.48–1.76)	0.79 (0.29–2.16)	1.04 (0.23–4.71)
History of allergy (ref. no history of allergy)		0.98 (0.69–1.39)	1.12 (0.81–1.56)	1.21 (0.9–1.64)	1.15 (0.81–1.63)	1.22 (0.65–2.3)	1.37* **(1.02–1.84)**	0.56 (0.31–1.01)	1.94*** **(1.45–2.6)**	1.39* **(1.04–1.87)**	1.08 (0.72–1.60)	1.13 (0.62–2.05)
History of anaphylactic shock (ref. no history of anaphylactic shock)		0.79 (0.2–3.08)	1.3 (0.41–4.1)	2.44 (0.85–6.96)	2.38 (0.8–7.03)	1.14 (0.13–9.64)	1.4 (0.5–3.95)	1.08 (0.13–8.69)	0.77 (0.27–2.18)	0.81 (0.29–2.27)	1.90 (0.57–6.35)	5.62** **(1.62–19.53)**
History of COVID-19 (ref. no history of COVID-19 )		2.04*** **(1.45–2.89)**	1.54** **(1.11–2.15)**	1.8*** **(1.33–2.43)**	2.04*** **(1.45–2.87)**	1.04 (0.55–1.99)	1.45* **(1.08–1.96)**	1.88* **(1.14–3.09)**	1.4* **(1.03–1.87)**	1.14 (0.84–1.52)	1.5* **(1.01–2.22)**	2.23** **(1.26–3.92)**
Type of vaccine: Comirnaty (ref. Jcovden)		0.15*** **(0.09–0.27)**	0.35*** **(0.23–0.53)**	0.63** **(0.45–0.88)**	0.78 (0.53–1.14)	5.44*** **(2.93–10.08)**	0.57*** **(0.41–0.79)**	0.79 (0.44–1.39)	1.02 (0.75–1.39)	0.71* **(0.52–0.96)**	0.68 (0.43–1.05)	0.8 (0.42–1.54)

Statistically significant correlations are set in bold; *p-value <0.05; **p-value ≤0.01; ***p-value ≤0.001; CI: confidence interval. OR: odds ratio. VAE: vaccine adverse event There was no statically relevant correlation of any variables and seizures, allergic reactions, presyncope, transient loss of consciousness, heart palpitation or other cardiovascular symptoms, diarrhoea, limb(s) discolouration, and vomiting. Table 4 presents only the variables with at least one statistically relevant correlation.

## Discussion

### Main findings

This study has analysed data from 1051 patients vaccinated with mRNAV (Comirnaty) or VVV (Jcovden). The vaccination proved safe in short-term observation; 8.8% of the participants reported neither local nor systemic VAEs. There were only 11 cases of transient loss of consciousness and two other hospital admission cases. Those receiving Jcovden were more likely to develop systemic reactions and less likely to have local symptoms when compared with the Comirnaty group. The history of anaphylactic reaction correlated with the increased risk of allergic reactions, nausea and cardiovascular symptoms. Female sex, younger age and a history of COVID-19 were the other predictors for experiencing systemic VAEs. Older and obese patients were less likely to report local reactions. Current cigarette smoking was a ‘protective’ factor for myalgia, headaches and excessive fatigue.

### Strengths and limitations

So far, this is one of the few studies to actively investigate VAEs in the primary care setting (data from an Australian large-scale system are already available [17], while those from Europe have not yet been published [18,19]). Our findings reflect the possible daily experience of primary care physicians vaccinating their patients against COVID-19. The other advantage of this project is that we conducted it during the national vaccination programme, when data on the safety of COVID-19 vaccination in the general population were still scarce. Given that, the bias risk in both participants and fieldworkers could be lower than in the case of retrospective interviews or analyses.

Contrary to the compulsory VAEs reporting system, we could identify patients with no or mild VAEs. In opposition to the studies recruiting volunteers, there was a lower risk of overreporting severe or multiple VAEs.

A specific limitation of the study is the relatively small number of patients and practices, as well as a limited geographical range. The low number of severe VAEs could result from general advice for persons with a history of severe allergic reactions to be vaccinated at nodal hospitals only. The other limitation is the small number of patients receiving Vaxzevria or Spikevax. That was beyond our control, as the central government agency supplied vaccination hubs, so the participating GP practices depended entirely on this system. Another disadvantage is that some of the data on VAEs after the first dose were collected retrospectively. However, the short period between the doses (4 weeks) makes the answers reliable. It is essential to underline that the presented results do not allow for reasoning about long-term VAEs.

### Comparison with existing literature

Our findings are consistent with other studies on the Polish population and with international reports on the safety of COVID-19 vaccines. Other studies’ findings on the selected groups in the Polish population [20–23] showed that COVID-19 vaccines’ VAEs are frequent but mild, similar to our results. Unlike Li et al. [24], we did not find a correlation between the history of allergy and any specific VAE and only a weak correlation with the number of systemic VAEs. More frequent reporting of VAEs by females or persons with a higher level of education could be partially explained by the differences in the attitude towards safety, as Syan et al. conclude in their study on the Canadian population [25]. Similar higher rates of VAEs were observed in females in Japan, but Urakawa et al. explain this with the role of sex hormones in immune response [26]. It is difficult to conclude whether the observed more frequent VAEs in younger patients depend on the type of vaccine or the age of the patients (those receiving VVV were younger than those vaccinated with mRNAV). However, Chen et al. in their meta-analysis and Wu et al. in their review found that VAEs were more frequent in the case of vaccination with VVV when compared with mRNAV. They also state that VAEs were more frequently observed in younger patients [27,28], also remarked by Urakawa et al. [26]. At some point, it is surprising that normal BMI compared with obesity correlated positively with the risk of generalised VAEs, yet it is consistent with the results of Iguacel et al. [29]. Similar to our observations, Tissot et al. identified a history of COVID-19 infection as a predictor of post-vaccination reactions [30].

### Recommendations for clinical practice and future research

Although the limitations do not allow one to generalise the findings, the study’s results might add evidence in the subject of COVID-19 vaccines’ safety. The real-life-based study’s results can help in discussions with hesitant patients and physicians. It also shows the need to maintain primary care research networks to facilitate data collection in GP practices covering broad areas and large populations. Pragmatic studies of the long-term safety of the vaccination might contribute to building a larger body of knowledge about the studied issues.

## Conclusion

We conclude that more than 90% of patients vaccinated against COVID-19 in primary care settings may experience VAEs in a short-term follow-up, and they are mostly mild. Their frequency is close to the manufacturers’ declarations but higher than reported by state institutions. Females, younger patients, those with higher education or a history of COVID-19 may experience systemic VAEs more frequently, while older and obese people are less likely to report local reactions.

## Supplementary Material

Supplemental Material
