# Supplementary material for: COVID-19 vaccine short-term adverse events in the real-life family practice in Krakow, Poland
Source: Eur J Gen Pract. 2022 Dec 5;29(2):2147500. doi: 10.1080/13814788.2022.2147500 (PMC10249448; doi:10.1080/13814788.2022.2147500)
Supplement: Supplemental Material [file IGEN_A_2147500_SM8336.docx]

Suplemental Materials

Suplemental Materials Table 1. Variables correlated with the number of local vaccine adverse events in the linear regression model

| Variable | β (SD) | 95%CI |  |
| --- | --- | --- | --- |
| Age  (ref. the lowest value) | -0.14 (0.04) | -0.21 to -0.07 |  |
| Obesity  (ref. normal BMI) | -0.18 (0.05) | -0.23 to -0.01 |  |
| Sex female  (ref. male) | 0.08 (0.03) | 0.02 to 0.15 |  |
| Chronic medical conditions (any)  (ref. no chronic conditions) | 0.1 (0.03) | 0.03 to 0.17 |  |
| History of COVID-19 infection  (ref. no history of COVID-19 infection) | 0.06 (0.03) | 0.002 to 0.12 |  |
| Type of vaccine: Comirnaty  (ref. Jcovden) | 0.14 (0.032) | 0.08 to 0.21 |  |
| BMI – body mass index, CI – confidence interval, SD – standard deviation | | | |

Suplemental Materials Table 2. Variables correlated with the number of systemic vaccine adverse events in the linear regression model

| Variable | β (SD) | 95%CI |
| --- | --- | --- |
| Age  (ref. the lowest value) | -0.18 (0.036) | -0.25 to -0.11 |
| Obesity  (ref. normal BMI) | -0.16 (0.055) | -0.26 to -0.05 |
| Sex female  (ref. male) | 0.15 (0.032) | 0.08 to 0.21 |
| Higher education  (ref. elementary education) | 0.07 (0.033) | 0.01 to 0.14 |
| Chronic medical conditions (any)  (ref. no chronic conditions) | 0.08 (0.03) | 0.01 to 0.14 |
| History of allergy (any)  (ref. no history of allergy | 0.09 (0.03) | 0.03 to 0.18 |
| History of COVID-19 infection  (ref. no history of COVID-19 infection) | 0.13 (0.03) | 0.07 to 0.19 |
| Type of vaccine: Comirnaty  (ref. Jcovden) | -0.13 (0.03) | -0.19 to -0.06 |
| BMI – body mass index, CI – confidence interval, SD – standard deviation | | |
